# Emergency Department Management of Low Back Pain: A Comparative Review of Guidelines and Practices

**DOI:** 10.7759/cureus.53712

**Published:** 2024-02-06

**Authors:** Alec M Werthman, Brayden D Jolley, Andrew Rivera, Melissa A Rusli

**Affiliations:** 1 Internal Medicine, Lakeland Regional Health Medical Center, Lakeland, USA

**Keywords:** chronic pain, opioid use, pain alleviation, treating low-back pain, chronic lower back pain, acute low back pain

## Abstract

This narrative review examines the current best practices and guidelines for integrating pharmacologic interventions, imaging, and physiotherapy in the management of low back pain. The review also explores how patient factors such as age, sex, comorbidities, and prevalent pathologies/diagnoses influence the choice and effectiveness of these treatment approaches.

## Introduction and background

Background

Low back pain represents a prevalent musculoskeletal concern on a global scale, imposing a significant burden on individuals, healthcare systems, and economies alike [1,2]. It has numerous etiologies but often arises from three more common causes: lumbosacral discomfort, radicular pain, and referred pain [3]. Further, these causes of low back pain can be dissected into more specific pathologies including myofascial discomfort, facet joint-related pain, sacroiliac joint issues, vertebrogenic pain, discogenic pain, spinal stenosis, and failed back surgery syndrome [3,4]. Importantly, back pain ranks among the primary motives prompting adults aged 18-65 years to seek emergency medical attention [5,6], and, within this broad patient population, there are multiple factors, such as age, sex, and other comorbidities, which have varied associations with back pain prevalence and etiology [1,3,7,8]. Despite an abundance of clinical guidelines for the diagnosis and management of low back pain, both in the acute setting and as a chronic disease, there remains significant evidence of widespread deviation from these guidelines [9,10]. Low back pain not only represents a persistent health concern but also imposes a considerable financial burden on the healthcare system, particularly in the United States; as such, optimizing its management offers a significant opportunity to enhance patient outcomes and alleviate healthcare costs [6,11].

Objective

This review aims to synthesize the current literature on the best practices for the management of low back pain in the adult population, focusing on pharmacologic interventions, imaging strategies, and physiotherapy. It also seeks to understand how patient-specific factors can influence treatment options and outcomes.

## Review

Pharmacologic interventions for low back pain

Agreement

The majority of the literature agrees on the efficacy of nonsteroidal anti-inflammatory drugs (NSAIDs) for low back pain management and recommends them as first-line agents [12]. Studies show that these medications are generally effective in the short term for alleviating pain, improving function, and decreasing length of stay for low back pain [13-15]. Recommendations are aligned in suggesting acetaminophen, NSAIDs, and muscle relaxants for short-term relief, while opioids are suggested only for severe refractory pain [13,14,16]. However, the studies were consistent in reporting frequent prescriptions of opioids in the ED for low back pain [17,18] and their detrimental effects on patient outcomes [19,20]. Extracorporeal pharmacologic interventions, chiefly topical lidocaine, are generally recommended for acute back pain and have, in some cases, demonstrated greater effectiveness than neuropathic medications [21]. ​

Inconsistencies

There was no consensus on the additional benefit of combining other medications with NSAIDs. One study reported decreased opioid prescribing for patients who received either acetaminophen or NSAIDs [18], while other studies found no benefit from acetaminophen for the treatment of low back pain [12,22]. There was a similar disagreement on the effectiveness of muscle relaxants. Some studies recommend the use of muscle relaxants [23,24] while other guidelines report ineffective pain control with muscle relaxants [25].

Gaps in Research

There is widespread agreement on the limited use of opioids and their detrimental long-term effects. Despite recommendations against first-line use of opioids, however, there is a broad acknowledgment of overprescription and chronic use of opioids for patients in emergency departments [18] and following discharge [26]. We found no consensus on the reason for this discrepancy. Likewise, there is a relative scarcity of high-quality studies that examine the long-term effects of medication therapy for low back pain, particularly in older adults and those with comorbidities. While one study reported no additional benefit from adding muscle relaxants to naproxen [25], we did not find any studies comparing their effectiveness as a single agent versus NSAIDs or acetaminophen. Data shows ketamine to be an effective opioid alternative for acute and chronic pain in emergency settings [27]. There is, however, a scarcity of data on the use of ketamine specifically for back pain as well as for repeated use across a variety of patient populations and conditions [28]. Few studies were found on the utility of topical medications other than lidocaine for back pain. Many topical interventions have generally been studied as a treatment for chronic pain outside of the emergency department, and often for dermatologic or post-viral neuropathic pain [29]. There is a significant opportunity to investigate topical medications for their utility as single agents for back pain presentations in emergency departments. Furthermore, our review did not find any data stratifying back pain severity, frequency, or management based on outpatient establishment prior to, or after, emergency presentation. Interventions performed in the emergency setting would likely be better contextualized based on the degree of pain management in the outpatient setting.

Diagnostic imaging in low back pain management

Agreement

Multiple guidelines and reviews concur that routine imaging is not recommended for acute-onset, atraumatic low back pain without concerning historical factors [5,13,14,30]. While plain radiography is commonly used as an initial imaging test, the evidence does not support its routine use, and, in multiple studies, the rates of patients receiving inappropriate imaging for a low back pain complaint was consistently found to be approximately one in three [9,11,17]. Interestingly, one study also found that some patients did not receive imaging despite the presence of clinical red flags such as cauda equina, cord compression, and infection, which expands on the concern for inappropriately adhering to imaging guidelines [10]. Guidelines broadly discourage routine use but specifically prescribe it in the presence of red-flag symptoms concerning for urgent pathology [13,14,30]. Beyond X-ray imaging, there is agreement that advanced imaging modalities, such as MRI or CT are not indicated for acute back pain but can be appropriate in certain clinical scenarios like surgery candidates or persistent subacute symptoms even in the absence of red flag symptoms [5,30]. Notwithstanding the extensive clinical recommendations, real-world data consistently shows poor utilization of imaging studies for low-back pain [9,10,17].

Inconsistencies

Some disagreement exists on the real-world use of advanced imaging for low back pain. While there is general agreement on the high rate of imaging relative to guidelines, varying estimates are reported on the frequency of CT and MRI use in the emergency department. Additionally, one of these studies found increasing use of CT/MRI over time [9], while another found decreasing use [17].

Gaps in Research

Several knowledge gaps persist around imaging for low back pain. Appropriate criteria and thresholds for advanced imaging in subacute or chronic low back pain require further delineation [5,16]. The impact of early imaging on outcomes like pain, function, and healthcare utilization needs clarification [23,30]. Real-world adherence to guidelines around avoiding routine acute imaging warrants further investigation [9,17], along with the cost-effectiveness of different strategies and comparative accuracy of modalities like radiography versus MRI [10,31]. However, given improving access to interventional radiographic procedures and their increasing frequency of use, recent data on the indications for advanced imaging in cases of low back pain could demonstrate a component of appropriate workup for newer interventional treatments.

Physiotherapy and alternative therapies

Agreement

Current clinical guidelines and literature reviews largely agree on the preferred management strategies for low back pain, recommending patient education and lifestyle modification such as remaining active as initial treatments rather than routine imaging or passive modalities [1,13,16,23,30]. Non-pharmacological therapies like exercise, physical therapy, spinal manipulation, heat and cold therapies, massage, acupuncture, mindfulness-based stress reduction, yoga, and cognitive behavioral therapy are also supported by multiple sources [14,32,33,34]. While there are some discrepancies between the sources, there is consensus around the value of patient education, lifestyle modification, and exercises as preferred strategies for managing low back pain. Trigger point injections (TPIs) are also effective for managing back pain resulting from myofascial trigger points. Whether used as a primary or complementary therapy, TPIs play a valuable role in reducing musculoskeletal pain in both outpatient and emergency department settings, reducing the length of stay and decreasing the need for opioids [35-37].

Inconsistencies

While there is consensus on many non-pharmacological management strategies, some inconsistencies exist regarding the efficacy of certain therapies. Specifically, there are conflicting results on acupuncture for low back pain, with some reviews recommending it [13,16], while guidelines recommend against its routine use [23]. There is also disagreement on the long-term benefits of spinal manipulation, with some sources finding sustained benefits [13,14] and others showing mixed results [16,23,30].

Gaps in Research

There is a lack of consensus around effective prevention strategies for reducing recurrent or persistent back pain as well as clear criteria concerning referral for advanced surgical evaluation. Further high-quality research is needed to investigate these gaps and inconsistencies in the non-pharmacological management literature in order to establish stronger evidence-based recommendations. More large-scale, high-quality studies are needed to assess the long-term efficacy and optimal combination of these non-pharmacologic interventions. There seems to be a gap in the literature regarding whether the adoption of alternative and physiologic therapies is associated with decreased repeated visits to emergency care. Osteopathic manipulative treatment (OMT) was reported to decrease the requirement for pharmacologic pain management and reduce the length of stay for musculoskeletal pain [38], but we found no data on the use of OMT in any large-scale studies or regarding its efficacy specifically in emergency departments.

Influence of patient factors

Agreement

The literature broadly agrees on the most common presentation of low back pain in the ED being a non-specific, chronic low back pain [1,11,39]. Further, it was widely found that ED presentation for low back pain was associated with a sedentary lifestyle, excess body weight, and chronicity of pain [7,8]. Older age was found to significantly influence management and increase the likelihood of admission as well as longer length of stay [7]. There is general recognition of psychosocial factors having a significant effect on chronicity and recurrence of pain. Furthermore, reports across the United States and Canada document an increasing rate of presentations to the ED for low-back pain [32,33].

Inconsistencies

Research varies on the significance of demographic factors in low back pain. Some studies found age and gender at low and high BMI to be predictive of back pain etiology, with internal disc disruption, facet joint pain, or sacroiliac joint pain as the most common cause [4,7]. It was otherwise found that age was more closely correlated with frequency of presentation and length of stay rather than etiology of back pain [40], and gender played no significant role [8].

Gaps in Research

While multiple studies have investigated the influence of demographic, psychosocial, and socioeconomic factors on low-back pain presentations to the ED, the dependent variables investigated shared little overlap. Additionally, in studies investigating the same outcome, the inclusion criteria for patients studied shared minimal overlap. Research into the influence of race and socioeconomic status on low back pain was conducted with limited parameters, focusing on outcomes in African American versus Caucasian patients and rates of admission for higher-income patients [41]. We also found no significant investigations on the influence of primary language on low back pain presentations to the ED. Given the growing Hispanic population and the prevalence of Spanish as a primary language in the United States, there is a significant gap in the research for these patients. Furthermore, studies broadly agree on an increasing rate of presentations to the ED for low back pain, yet no evidence-based explanations or recommended interventions are given to combat this increase.

Discussion

The aim of this narrative review was to synthesize the existing literature on the management of low back pain in adult patients, with a focus on pharmacological interventions, diagnostic imaging, physiotherapy, and the influence of patient-specific factors. While a number of commonalities emerged from the reviewed studies, such as the first-line use of NSAIDs for pain management and the general discouragement of routine imaging, significant variations and gaps in current practice and knowledge were also noted.

Pharmacologic Interventions

Consensus predominantly favors the use of NSAIDs as first-line pharmacological agents for managing low back pain. This concurrence points to the broad acceptance and effectiveness of NSAIDs in alleviating acute symptoms. However, the persistent over-prescription of opioids is a concerning discrepancy, given the associated risks of long-term use and dependency. The lack of high-quality research into the long-term outcomes of medication therapy, especially among older adults and those with comorbidities, highlights an area that warrants immediate attention.

Diagnostic Imaging

The general agreement against routine imaging for uncomplicated low back pain signifies a move toward more evidence-based practice. Nonetheless, the real-world data indicate an alarming deviation from this, with inappropriate use of imaging in a substantial proportion of cases. This discord raises questions about the effectiveness of current guidelines and suggests the need for more robust education or systems-based solutions to ensure adherence.

Physiotherapy and Alternative Therapies

Despite the divergence in views on the efficacy of some non-pharmacological therapies like acupuncture and spinal manipulation, the literature broadly supports patient education, lifestyle modification, and a range of alternative therapies as effective initial strategies. The gaps identified in preventive strategies and long-term outcomes offer avenues for further research.

Influence of Patient Factors

While the literature commonly identifies chronic low back pain as the most prevalent type presenting in emergency departments, the influence of demographic factors remains less consistent across studies. Future research could benefit from standardized inclusion criteria to facilitate more meaningful comparisons. Moreover, the limited scope of studies focusing on the influence of race, socioeconomic status, and primary language reflects a research gap that is particularly relevant given the demographic diversity of the United States.

Limitations and future directions

A key limitation of this review is the inclusion of only studies published within the last 20 years, which may exclude relevant findings from earlier research. Additionally, the focus on adult patients presenting in EDs may not be fully generalizable in settings with different systems of triage or across all age groups.

Moving forward, it is imperative to conduct more high-quality, long-term studies to bridge the identified gaps in understanding the most effective strategies for low back pain management. This could include randomized controlled trials comparing the long-term efficacy of pharmacological and non-pharmacological treatments, investigations into the reasons for poor adherence to imaging guidelines, and studies focused on understudied demographic groups.

## Conclusions

The management of low back pain remains a complex and multi-faceted challenge that continues to burden healthcare systems, particularly in the United States. This review highlights both the advancements in understanding effective treatments and the gaps that need to be addressed in order to provide more comprehensive and individualized care. While significant progress has been made in delineating effective management strategies, substantial work remains to optimize the alignment between evidence-based guidelines and real-world practice.

By filling the identified research gaps and minimizing inconsistencies in practice, we may move closer to a more standardized, effective, and cost-efficient management approach for low back pain, ultimately benefiting both individual patients and healthcare systems at large.
